# Gender-specific associations of pregnancy-related anxiety with placental epigenetic patterning of glucocorticoid response genes and preschooler’s emotional symptoms and hyperactivity

**DOI:** 10.1186/s12887-021-02938-z

**Published:** 2021-10-29

**Authors:** Hui Liu, Yuwei Liu, Kun Huang, Shuangqin Yan, Jiahu Hao, Peng Zhu, Fangbiao Tao, Shanshan Shao

**Affiliations:** 1 Department of Maternal, Child and Adolescent Health, School of Public Health, Anhui Medical University, No 81 Meishan Road, Hefei, 230032 Anhui China; 2 Key Laboratory of Population Health Across Life Cycle (Anhui Medical University), Ministry of Education of the People’s Republic of China, No 81 Meishan Road, Hefei, 230032 Anhui China; 3 NHC Key Laboratory of Study on Abnormal Gametes and Reproductive Tract, No 81 Meishan Road, Hefei, 230032 Anhui China; 4 Anhui Provincial Key Laboratory of Population Health and Aristogenics, Anhui Medical University, No 81 Meishan Road, Hefei, 230032 Anhui China; 5 Maternal and Child Health Care Center of Ma’anshan, No 24 Jiashan Road, Ma’anshan, 243011 Anhui China

**Keywords:** Pregnancy-related anxiety, Child neurobehavior, Gender, Glucocorticoid, Placenta

## Abstract

**Backgroud:**

We have recently reported that maternal prenatal pregnancy-related anxiety predicts preschoolers’ emotional and behavioral development in a gender-dependent manner. This study aims to test for this gender-specific effect in a different cohort and investigate whether the gender difference was specific to placental methylation of genes regulating glucocorticoids.

**Methods:**

A total of 2405 mother–child pairs from the Ma’anshan Birth Cohort Study were included in present study. The maternal pregnancy-related anxiety symptoms were evaluated with the Pregnancy-Related Anxiety Questionnaire in the third trimester of pregnancy. Child neurobehavior was assessed with the Strengths and Difficulties Questionnaire at 4 years old. Placental methylation of *FKBP5*, *NR3C1* and *HSD11B2* genes was quantified using the MethylTarget approach in 439 pregnant women. After exploratory factor analysis, the associations between methylation factor scores and pregnancy-related anxiety and child neurobehavior were examined using logistic regression analysis.

**Results:**

After controlling for confounding factors, pregnancy-related anxiety in the third trimester of pregnancy increased the risk of hyperactivity only in boys and emotional symptoms only in girls. Decreased scores of the factor characterized by *FKBP5* methylation were associated with maternal pregnancy-related anxiety only in boys. Furthermore, increased scores of the factors characterized by *NR3C1* and *HSD11B2* methylation were associated with hyperactivity (*NR3C1*: adjusted OR = 1.80, 95%CI = 1.15–2.83) and emotional symptoms (*HSD11B2*: adjusted OR = 0.53, 95%CI = 0.29–0.97; *NR3C1*: adjusted OR = 1.64, 95%CI = 1.03–2.59) only in boys. However, the scores of the factor characterized by *FKBP5, NR3C1* and *HSD11B2* did not mediate the relationship between maternal pregnancy-related anxiety and preschoolers’ emotional symptoms and hyperactivity.

**Conclusions:**

Our results suggested that pregnancy-related anxiety in the third trimester of pregnancy predicted preschoolers’ emotional symptoms and hyperactivity in a gender-dependent manner. Although we did not find the mediation role of the placental methylation of genes regulating glucocorticoids, we found it was associated with both maternal pregnancy-related anxiety and preschoolers’ emotional symptoms and hyperactivity in a gender-dependent manner.

**Supplementary Information:**

The online version contains supplementary material available at 10.1186/s12887-021-02938-z.

## Backgroud

Pregnancy-related anxiety, a specific form of prenatal maternal psychological stress (PNMS), affects 11–25% of pregnant women in the world [1, 2]. It refers to maternal unique psychological pressure and physical symptoms on baby’s well-being, their own health and appearance and also medical, economic and social issues in the context of pregnancy, childbirth and parenting [3]. Our group recently published two papers showing that pregnancy-related anxiety, as with other modes of PNMS (i.e. life events [4], catastrophic events [5] and anxiety not specific to pregnancy [6]), could predict child emotional and behavioral problems in a gender-specific pattern: conduct disorder and hyperactivity only in boys and emotional problems only in girls [7, 8]. The underlying mechanism of gender differences in the effect of PNMS on child development is still poorly understood.

However, the mechanism of neurobehavioral development induced by stress during pregnancy is relatively clear. Many studies have shown that fetal exposure to excessive glucocorticoids is considered to be an important intrauterine environment change of PNMS affecting the offspring’s psychopathology [9, 10]. Furthermore, changes in the intrauterine environment caused by PNMS may influence the genetic (or epigenetic) background of the fetus, shaping the developmental trajectory of the offspring’s neurobehavioral outcomes [11–13]. The placenta as the maternal–fetal intermediary is responsible for regulating the passage of maternal hormones into the uterine environment by expressing genes regulating glucocorticoids [14], including *HSD11B2* (encoding 11β-HSD2, an important enzyme that degrades glucocorticoids), *NR3C1* (encoding the glucocorticoid receptor) and *FKBP5* (encoding the FK506 binding protein that regulates expression of the glucocorticoid receptor [15, 16]. In recent years, researchers have found that multiple types of prenatal stress (i.e. anxiety, depressive, chronic and war-related stressors) can predict the methylation of the above-mentioned glucocorticoid response genes in neonatal cord blood, placenta and maternal blood [17–19]; and this methylation of the glucocorticoid response genes was found to be associated with early neurobehavioral development in infants [11]. Furthermore, Conradt et al. found that the effects of maternal depression or anxiety during pregnancy on newborn neurobehavior depended on the DNA methylation of placental genes *HSD11B2* and *NR3C1* [20].

In addition, several reviews in recent years have suggested that placental signal transduction induced by prenatal stress is likely to be gender-dependent [14, 21, 22]. Available evidence from animal models has also suggested that the placental gene expression response to prenatal stress signals may be in a gender-dimorphic pattern [23, 24]. Specifically, Challis et al. found that expression of 11β-HSD2 mRNA was elevated significantly in the placenta of male fetuses whose mothers had received dexamethasone in early pregnancy [25]. However, Stark et al. found that expression of 11β-HSD2 was higher in placenta of female fetuses born within 72 h following antenatal dexamethasone [26]. Pierre et al. suggested that prenatal stress from a natural disaster did not affect placental 11β-HSD2 mRNA [27]. These existing evidence of gender-dimorphic pattern of placental gene expression is inconsistent, further research is required.

Since the social and biological background related to PNMS varies in different countries [28, 29], the gender-specific response of placental gene expression to PNMS signals requires further investigation, especially in the middle or low income countries. The aim of our study was to verify previous findings in a new cohort and explore possible placental epigenetic mechanisms related to glucocorticoid uptake and inactivation genes in China. We hypothesized that exposure to maternal pregnancy-related anxiety in utero may program child gender-specific neurobehavior via gender-specific DNA methylation of *HSD11B2*, *NR3C1* and *FKBP5* genes in placenta.

## Methods

### Participants

Our participants were from the Ma’anshan Birth Cohort (MABC) study, a population-based prospective study that recruited pregnant women during early pregnancy in the city of Ma’anshan, China, from May 2013 until September 2014. Inclusion criteria for this cohort were as follows: ≤14 gestational weeks; ≥18 years old; living in Ma’anshan city for more than 6 months; planning on delivering at the Maternal and Child Health Care Centre of Ma’anshan; and good communication and interpersonal skills. A total of 3474 pregnant women who met the inclusion criteria were included in this cohort and followed-up for their physical and mental health information at the second and third trimesters of pregnancy. Postpartum, 3273 single live-birth children were invited for follow-up to assess their development and growing environment postnatally at: 0 and 42 days; 3, 6, 9, 12 and 18 months; and 3, 4 and 5 years. A total of 2405 (69.2%) mother–child pairs who completed both the assessment of pregnancy-related anxiety at the third trimester and emotional symptoms and hyperactivity at 4 years old were included in this study. Comparison of the basic maternal characteristics between those who were recruited in the final data analysis and those who dropped out is shown in Supplementary Table 1. This study was approved by the ethics committee of Anhui Medical University (Reference number: 20180084) and written informed consent was obtained from each pregnant woman.

### Pregnancy-related anxiety

Pregnancy-related anxiety in the third trimester of pregnancy was measured using the Pregnancy-Related Anxiety Questionnaire, which comprised 13 items across three subscales: “fear of woman’s own health” (six items); “fears related to the health of the fetus” (five items); and “fear of childbirth” (two items). Participants were required to rate their answers on a four-point Likert scale from 1 (never worried) to 4 (always worried). Scores of all the items were summed, with a total score of 13–52; a higher score indicates a higher level of anxiety. The pregnant woman whose score reached or exceeded the 75th percentile of the total score will be evaluated as having pregnancy-related anxiety. The scale was given in Chinese, which was developed by our team based on the Chinese population and has been verified in 7017 pregnant women in Anhui Province, China, with Cronbach’s alpha of 0.81 and a test–retest reliability of 0.79 [30].

### Children’s emotional symptoms and hyperactivity

Children’s emotional symptoms and hyperactivity were evaluated by the Strengths and Difficulties Questionnaire (SDQ), which is a brief behavioral screening instrument used to measure emotional and behavioral difficulties and prosocial behavior of 4–16-year-olds [31]. The SDQ contained 25 items and covered five subscales relating to the child’s emotional symptoms, conduct problems, hyperactivity, peer relationship problems and prosocial behavior. Each subscale consists of five questions rated on a three-point Likert scale (not true = 0; somewhat true = 1; certainly true = 2) and scores in the range 0–10. Higher scores represent greater symptom severity. In our study, we used the Emotional Symptoms and Hyperactivity subscales of the SDQ, which was filled out by parents (91.6%), grandparents (7.6%) or others (0.8%) of the child. Children were assessed at a mean of 48.96 months (standard deviation was 2.70 months). The preschooler whose subscale score is above the 80th percentile (indicating borderline and abnormal) will be identified as having problems on this subscale [32]. Specifically, the cut-off scores in our study were 3 and 6, respectively. SDQ used in our study was given in Chinese, translated from English. As described in Du et al. 2008 [33], the Cronbach’s α coefficients for the Emotional Symptoms and Hyperactivity subscales of the Chinese translation of the SDQ were 0.60 and 0.76, respectively. Besides, the convergent and discriminant validity proved to be acceptable [33].

### CpG islands selection, sample collection and DNA methylation detection

**Fig. 1:**
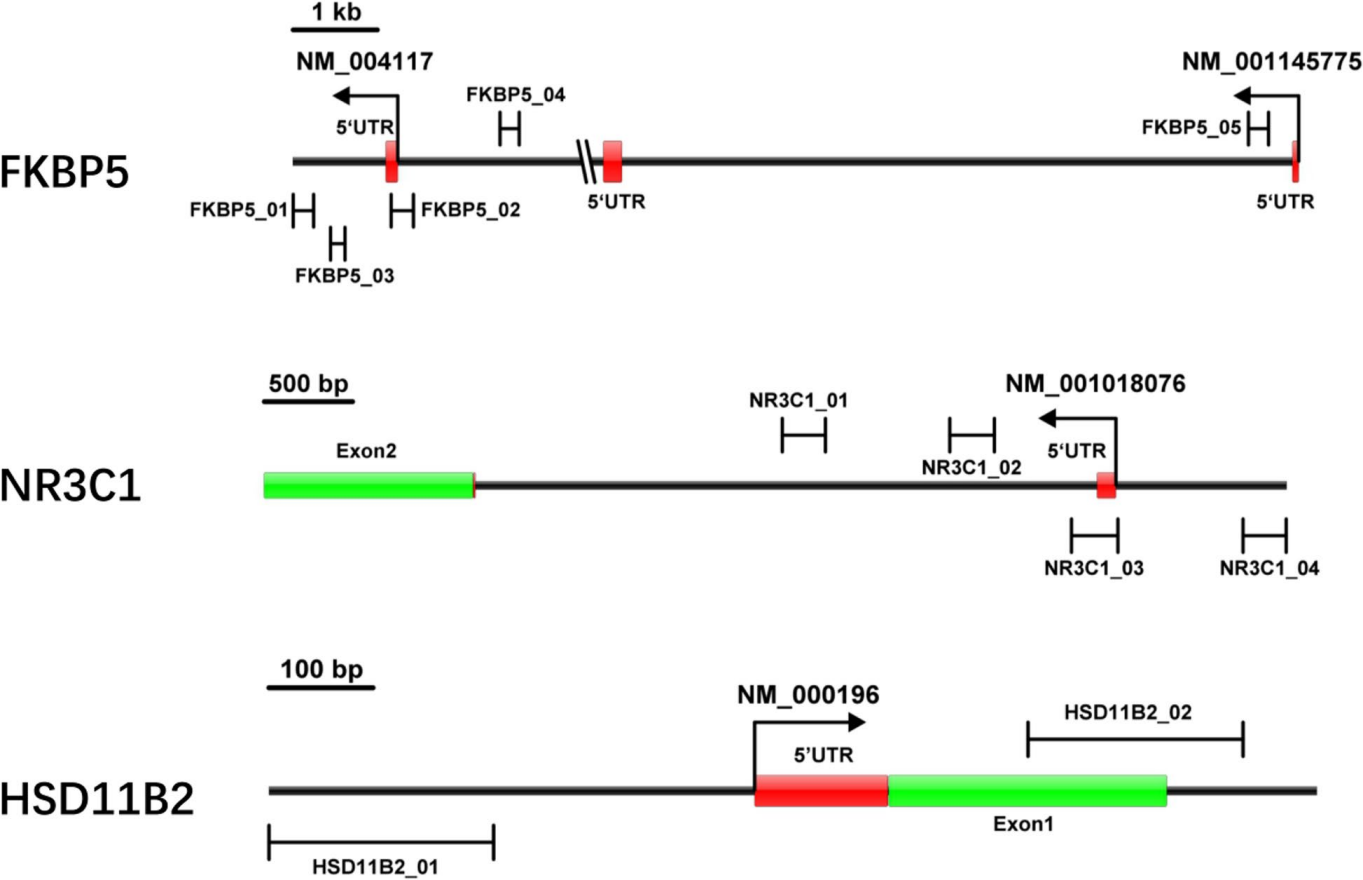
Regions from CpG islands of *FKBP5*, *NR3C1* and *HSD11B2. FKBP5_01* contains 11 CpGs; *FKBP5_02* contains 35 CpGs; *FKBP5_03* contains 20 CpGs; *FKBP5_04* contains 13CpGs; *FKBP5_05* contains 25 CpGs. *NR3C1_01* contains 27 CpGs; *NR3C1_02* contains 35 CpGs; *NR3C1_03* contains 29 CpGs; *NR3C1_04* contains 20 CpGs; *HSD11B2_01* contains 20 CpGs; *HSD11B2_02* contains 28 CpGs

We selected CpG islands located in the promoter of the *FKBP5*, *NR3C1* and *HSD11B2* genes from 2 kb upstream of the transcriptional start site (TSS) to 1 kb downstream of the first exon according to the following criteria [34]: (1) 200 bp minimum length; (2) 50% or higher cytosine–guanine content; (3) 0.60 or higher ratio of observed/expected CpG dinucleotides. Finally, four regions from CpG islands of the *NR3C1* gene (111 CpG sites), two regions from CpG islands of the *HSD11B2* gene (48 CpG sites) and five regions from CpG islands of the *FKBP5* gene (104 CpG sites) were selected and sequenced (Fig. 1).

Placental lobules from the full-thickness placenta 5 cm around the umbilicus were collected by trained personnel within minutes after delivery. The placental lobules were then immediately snap-frozen in liquid nitrogen, transported to the laboratory within 24 h and preserved at − 80 °C until further analysis. The 2405 subjects included were ranked in descending order according to the Pregnancy-Related Anxiety Questionnaire scores. The placentas of the top 300 subjects (pregnancy-related anxiety group) and the bottom 300 subjects (control group with no pregnancy-related anxiety) were then analyzed for DNA methylation.

Genomic DNA was extracted from the placenta tissue of the above two groups using a QIAGEN kit (QIAGEN, Hilden, Germany). DNA methylation detection was performed using MethylTarget™ (Genesky Biotechnologies Inc., Shanghai, China). In brief, the procedure was as follows: DNA was subjected to sodium bisulfite treatment using an EZ DNA Methylation™-GOLD kit (Zymo Research) according to the manufacturer’s instructions; multiplex polymerase chain reaction (PCR) was performed with an optimized primer set combination; PCR amplicons were diluted and amplified using indexed primers; index PCR amplicons were separated by agarose gel electrophoresis and purified using a QIAquick gel extraction kit (QIAGEN); and libraries from different samples were quantified and pooled together, followed by sequencing on the Illumina NextSeq platform according to the manufacturer’s instructions. Sequencing was performed with a 2 × 150 bp paired-end mode. The methylation levels of each CpG were equal to the ratio of methylated cytosine to total cytosine.

### Confounding factors

Based on existing literature and the results of our univariate analysis, we considered the following variables as confounders: maternal age, pre-pregnancy body mass index (BMI), gestational weight gain, education, family monthly income, smoking, drinking, gestational diabetes, pregnancy-induced hypertension, delivery mode and exclusive breastfeeding in the first 6 months. Information on maternal age, pre-pregnancy BMI, education, family monthly income, smoking and drinking was obtained through self-assessment questions in the first trimester questionnaire. Pregnancy complications, including gestational diabetes, pregnancy-induced hypertension and child gender, birthweight and gestational age at delivery were extracted from medical records. Information on gestational weight gain and feeding patterns at 6 months was derived from the postnatal questionnaire filled out by parents or other caregivers of preschoolers. The distribution of the covariates is shown in Table 1.

**Table 1:** Characteristics of participants (*n* = 2405)

	Prenatal anxiety	Control	*χ*^*2*^*/t*	*P*
Maternal age, years	26.21 ± 3.32	26.81 ± 3.70	3.95	**0.000**
Pre-pregnancy BMI^a^	20.50 ± 2.73	20.62 ± 2.78	1.01	0.311
Gestational weight gain^a^	18.13 ± 5.01	17.55 ± 5.07	−2.55	**0.011**
Maternal education			11.20	**0.011**
Bachelor degree or above	163(22.9)	478(28.3)		
Junior college	217(30.4)	536(31.7)		
Senior high school or equal	182(25.5)	360(21.3)		
Junior high school or below	151(21.2)	318(18.8)		
Family monthly income			5.74	0.057
< 2500 RMB	222(31.2)	448(26.5)		
2500–4000 RMB	294(41.2)	726(42.9)		
> 4000 RMB	197(27.6)	518(30.6)		
Maternal smoking			10.45	**0.001**
Never	670(94.0)	1638(96.8)		
Former/current	43(6.0)	54(3.2)		
Maternal drinking			1.20	0.274
Never	651(91.3)	1567(92.6)		
Occasionally/frequently	62(8.7)	125(7.4)		
Gestational diabetes	80(11.2)	215(12.7)	2.73	0.435
Pregnancy-induced hypertension^a^	28(3.9)	72(4.3)	2.20	0.532
Preterm birth	17(2.4)	58(3.4)	1.81	0.179
Low birth weight^a^	12(1.7)	34(2.0)	0.37	0.831
Cesarean^a^	389(2.4)	819(3.4)	7.83	**0.005**
Boys	360	874	2.27	0.602
Exclusive breastfeeding at first 6 months^a^	59(8.5)	215(13.0)	9.64	**0.002**

Abbreviations: *BMI* body max index, *RMB* Chinese monetary unit *yuan*

^a^The survey data is missing

### Statistical analysis

The participants’ characteristics are presented as mean ± standard deviation (mean ± SD) or number (frequency). Differences in the distribution of demographic characteristics between the pregnancy-related anxiety group and the control group were assessed using the *t*-test for continuous variables and chi-square tests for proportions. Logistic regression models were used to estimate the odds ratio (OR) with 95% confidence interval (95% CI) in the relationship between maternal pregnancy-related anxiety in the third trimester and children’s emotional symptoms and hyperactivity. The OR and 95% CI values were adjusted for several confounding factors, including maternal age, pre-pregnancy BMI, gestational weight gain, education, family monthly income, smoking, drinking, gestational diabetes, pregnancy-induced hypertension, delivery mode and exclusive breastfeeding in the first 6 months.

In this study we used factor analysis, which described variability between 263 CpG sites as a lower number of latent factors, to reduce the number of comparisons made. Factor analysis uses factor rotation of maximize orthogonal rotation (maximum variance method). Factor significance is defined by eigenvalues of > 2. Factor load, representing the correlation between individual CpG methylation and factor scores, was used to determine the contribution of individual CpGs to each factor; CpGs with an absolute factor loading of ≥0.3 were retained. Logistic regression was used to investigate the relationship between potential methylation variables generated by factor analysis and pregnancy-related anxiety in the third trimester and children’s emotional and hyperactivity stratified by infant gender. Then, the PROCESS program of mediation was used to perform a mediation analysis [35]. To test the mediating roles of potential methylation variables in the relationship between pregnancy-related anxiety in the third trimester and children’s emotional and hyperactivity. This approach uses bootstrapping to estimate all of the parameters. The mediating effect was tested using a bootstrap estimation approach with 5000 repetitions. When the 95% CI did not contain 0, the indirect effect was considered significant.

We also performed sensitivity analyses to check the robustness of our results. First, we excluded preterm birth (gestation < 37 weeks). Maternal anxiety in the third trimester is associated with premature delivery [36], and premature infants have increased risk of long-term neurodevelopmental problems [37]. To the extent that the unmeasured pathology that triggers preterm birth also harms the fetus directly, preterm birth can be confused with neonatal outcome. Direct adjustment of gestational age as a mediating variable will lead to bias when analyzing the relationship between risk factors and neonatal outcome. Second, the interaction between the severity of birthweight and maternal anxiety had significant impact on infant development [38]. Direct adjustment of birthweight can cause bias, therefore we did not adjust for birthweight in the main analysis but carried out sensitivity analysis instead.

All statistical analyses were performed with SPSS 23.0 software. The level of significance was *P* < 0.05.

## Results

### Maternal demographic characteristics in relation to pregnancy-related anxiety

The demographic characteristics of the subjects are shown in Table 1. Of the 2405 participants, 713 (29.6%) had pregnancy-related anxiety in the third trimester, and their mean age was 26.21 ± 3.32 years. Women who experienced pregnancy-related anxiety in the third trimester were significantly younger, had more gestational weight gain, a low education level, smoked more and had lower rates of caesarean and exclusive breastfeeding at 6 months compared with those having no pregnancy-related anxiety. However, there was no significant difference in either group with regard to maternal pre-pregnancy BMI, family monthly income, drinking, gestational diabetes, pregnancy-induced hypertension, preterm birth, low birthweight or fetal gender. After stratifying by the gender of the infants, the boys’ mothers’ age, gestational weight gain and rates of caesarean and exclusive breastfeeding at 6 months were significantly different between the pregnancy-related anxiety group and the control group; among the girls, the maternal age, education level, smoking and rates of exclusive breastfeeding at 6 months were significantly different between the two groups. Details are shown in Supplementary Table 2. Note, although the difference in age is statistically significant, the mean difference is not clinically meaningful.

### Gender-specific associations between maternal pregnancy-related anxiety and emotional symptoms and hyperactivity

The gender difference between maternal pregnancy-related anxiety and emotional symptoms and hyperactivity in their infants is shown in Table 2. In 2045 mother–child pairs, 416 children were above the significant cut-offs for normal emotional symptoms. The prevalence of emotional symptoms was 16.9% (209/1234) for boys and 17.7% (207/1171) for girls. In addition, 393 children were hyperactive. The prevalence of hyperactivity was significantly higher in boys 19.4% (239/1234) than in girls 13.2% (154/1171).

**Table 2:** Gender-specific associations between maternal pregnancy-related anxiety during the third trimester and emotional symptoms, hyperactivity in 4 years old children

Prenatal-related anxiety	Emotional Symptoms				Hyperactivity			
	Normal	Borderline and Abnormal	OR^a^(95%CI)	*P*^a^	Normal	Borderline and Abnormal	OR^a^(95%CI)	*P*^a^
Boys								
No	735(84.1)	139(15.9)	1.00		724(82.8)	150(17.2)	1.00	
Yes	290(80.6)	70(19.4)	1.21(0.86,1.69)	0.269	271(75.3)	89(24.7)	1.54(1.13,2.11)	**0.007**
Girls								
No	695(85.0)	123(15.0)	1.00		719(87.9)	99(12.1)	1.00	
Yes	269(76.2)	84(23.8)	1.61(1.15,2.24)	**0.005**	298(84.4)	55(15.6)	1.13(0.76,1.66)	0.551

Abbreviations: *CI* confidence interval, *OR* odds ratio

^a^ represents that these ORs adjusted by maternal age, pre-pregnancy BMI, Gestational weight gain, education, family monthly income, smoking, drinking, gestational diabetes, pregnancy-induced hypertension, delivery mode and exclusive breastfeeding at first 6 months

In the total sample there was no interaction between pregnancy-related anxiety and infant gender for the risk of emotional symptoms (*P* for interaction = 0.122) and hyperactivity (*P* for interaction = 0.214); after controlling for confounding factors and compared with the control group, pregnancy-related anxiety in the third trimester increased the risk of preschool emotional symptoms (OR = 1.41, 95% CI = 1.12–1.78) and hyperactivity (OR = 1.33, 95% CI = 1.05–1.70). After stratifying by gender, in boys, mothers experiencing pregnancy-related anxiety showed an increased risk of having a child with hyperactivity (adjusted OR = 1.54, 95% CI = 1.13–2.11) compared with the non-anxious women; for girls, pregnancy-related anxiety in the third trimester was related to preschool girls’ emotional symptoms (adjusted OR = 1.61, 95% CI = 1.15–2.24). The sensitivity analysis was limited to children born full term and after adjusting for birthweight the results remained significant.

In this study, a total of 439 pregnant women’s placental tissues passed methylation quality tests. We Reanalyzed the relationship between prenatal-anxiety and child emotional symptoms/hyperactivity only the subset of participants (*n* = 439) with DNA methylation data. As shown in Supplementary Table 3, compared with the control group, pregnancy-related anxiety increased the risk of preschool hyperactivity (OR = 1.89, 95% CI = 1.00–3.58) after controlling for confounding factors. After stratifying by gender, these results indicated that there are no obviously significant association between maternal pregnancy-related anxiety and child emotional symptoms and hyperactivity at both genders.

### Factor analysis defines five factors that explain variability in methylation

Of the 263 CPG sites selected, 52 CpG sites with an average percentage methylation level of > 2% remained for statistical analysis [19].

**Fig. 2:**
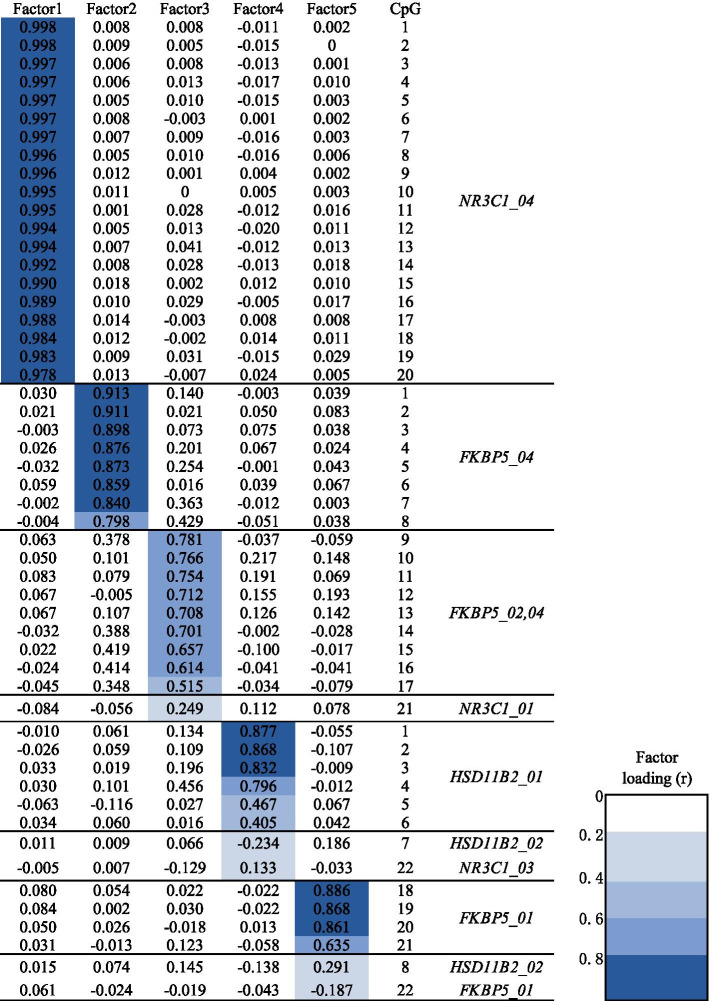
Loading of individual CpG onto latent methylation variables. The loading of each of the 52 CpGs across 5 latent factors was shown. Factor loadings greater than 0.3 were considered significant

Factor analysis shows that KMO (Kaiser-Meyer-Olkin) = 0.961 (*P* = 0.000) in Bartlett’s test of sphericity. Using eigenvalues of > 2 and a Scree plot, five significant orthogonal factors were extracted and their cumulative contribution rate was 73.8%. Figure 2 shows the rotated component matrix of five factors and the loadings of each CpG onto individual factors; a correlation of > 0.3 between the factor score and methylation of an individual CpG was considered a significant loading. *NR3C1* CpGs 1–20 were significantly loaded with factor 1, which explained 38.1% of the proportional variation in methylation, with a characteristic eigenvalue of 19.82. Factor 2 explained 17.6% of the proportional variation in methylation, with an eigenvalue of 9.15, and was significantly loaded by methylation of *FKBP5* CpGs 1–8. Factor 3 was significantly loaded by *FKBP5* CpGs 9–17 and explained 7.6% of the proportional variation in methylation, with an eigenvalue of 3.96. *HSD11B2* CpGs 1–6 and *FKBP5* CpGs 18–21 were significantly loaded with factors 4 and 5, respectively: 5.9 and 4.5% of the proportional variation in methylation was explained, with eigenvalues of 3.07 and 2.38, respectively. Each factor is only loaded with the CpG of a single gene. Thus, the five factors contain 47 CpGs. *NR3C1* CpGs 21–22, *HSD11B2* CpGs 7–8 and *FKBP5* CpG 22 did not load onto any factor and were not included in the analysis.

### Methylation of glucocorticoid regulation genes and maternal pregnancy-related anxiety in the third trimester and children’s emotional symptoms and hyperactivity

Of the 600 samples, 161 failed the methylation quality test and therefore placental tissue from 439 pregnant women was analyzed. Table 3 shows the association between latent methylation factors and pregnancy-related anxiety in the third trimester. In the total sample we observed that latent factor 5 was associated with lower risk of women who had pregnancy-related anxiety in the third trimester (OR = 0.80, 95% CI = 0.66–0.97). After controlling for confounding factors, the result remained significant (OR = 0.74, 95% CI = 0.59–0.92). In boys, latent factor 5 also decreased the risk of maternal pregnancy-related anxiety (adjusted OR = 0.62, 95% CI = 0.44–0.88). In girls, however, all the latent methylation factors were not associated with pregnancy-related anxiety. We found that factor 5 was characterized by *FKBP5* gene methylation*.* These results were largely unchanged after sensitivity analysis.

**Table 3:** Binary regression models for the association between pregnancy-related anxiety in the third trimester and latent methylation factors

Predictors	OR(95%CI)	*P*	OR^a^(95%CI)	*P*^a^
Boys				
Factor1^*^	1.01(0.76,1.35)	0.934	1.11(0.79,1.57)	0.558
Factor2^*^	1.16(0.86,1.55)	0.327	1.21(0.86,1.69)	0.274
Factor3^*^	1.00(0.76,1.31)	0.998	1.04(0.76,1.42)	0.813
Factor4^*^	1.19(0.89,1.59)	0.240	1.30(0.94,1.81)	0.115
Factor5^*^	0.68(0.51,0.91)	**0.008**	0.62(0.44,0.88)	**0.006**
Girls				
Factor1^*^	0.95(0.74,1.21)	0.655	0.96(0.71,1.31)	0.800
Factor2^*^	1.03(0.80,1.32)	0.835	1.10(0.83,1.46)	0.512
Factor3^*^	0.84(0.65,1.10)	0.201	0.92(0.67,1.25)	0.576
Factor4^*^	1.00(0.78,1.28)	0.970	1.19(0.89,1.59)	0.250
Factor5^*^	0.94(0.71,1.24)	0.658	0.92(0.66,1.28)	0.624

Abbreviations: ^*^ Latent methylation variables

^a^ represents that these ORs were calculated by binary logistic regression, adjusted by maternal age, pre-pregnancy BMI, gestational weight gain, education, family monthly income, smoking, drinking, gestational diabetes, pregnancy-induced hypertension, delivery mode and exclusive breastfeeding at first 6 months

Table 4 shows the association between latent methylation factors and emotional symptoms and hyperactivity in 4-year-old children. After stratifying by gender of the infants, latent factor 1 increased the risk of preschool boys’ emotional symptoms (adjusted OR = 1.64, 95% CI = 1.03–2.59) and hyperactivity (adjusted OR = 1.80, 95% CI = 1.15–2.83), whereas latent factor 4 decreased the risk of preschool boys’ emotional symptoms (adjusted OR = 0.53, 95% CI = 0.29–0.97). We found that factors 1 and 4 were characterized by *NR3C1* and *HSD11B2* gene methylation, respectively. These results were largely unchanged after sensitivity analysis.

**Table 4:** Binary logistic regression models for the association between the latent methylation factors and emotional symptoms and hyperactivity in 4 years old children

Predictors	Emotional Symptoms		Hyperactivity	
	OR^a^(95%CI)	*P*^a^	OR^a^(95%CI)	*P*^a^
Boys				
Factor1^*^	1.64(1.03,2.59)	**0.037**	1.80(1.15,2.83)	**0.011**
Factor2^*^	1.31(0.78,2.20)	0.300	1.22(0.76,1.95)	0.416
Factor3^*^	0.67(0.41,1.10)	0.112	1.04(0.68,1.60)	0.866
Factor4^*^	0.53(0.29,0.97)	**0.039**	0.89(0.57,1.38)	0.597
Factor5^*^	0.99(0.68,1.44)	0.936	0.67(0.44,1.03)	0.069
Girls				
Factor1^*^	1.05(0.69,1.58)	0.829	1.09(0.70,1.69)	0.710
Factor2^*^	0.71(0.46,1.09)	0.113	1.35(0.80,2.28)	0.258
Factor3^*^	0.94(0.60,1.47)	0.773	0.73(0.44,1.19)	0.206
Factor4^*^	1.21(0.79,1.85)	0.385	1.15(0.73,1.82)	0.543
Factor5^*^	0.93(0.57,1.52)	0.785	1.05(0.62,1.78)	0.855

Abbreviations: ^*^ Latent methylation variables

^a^ represents that these ORs were calculated by binary logistic regression, adjusted by maternal age, pre-pregnancy BMI, gestational weight gain, education, family monthly income, smoking, drinking, gestational diabetes, pregnancy-induced hypertension, delivery mode and exclusive breastfeeding at first 6 months

Sex-specific mediating role of the latent methylation factors 1, 4 and 5 in the relationship between maternal pregnancy-related anxiety and children’s emotional symptoms and hyperactivity was conducted. As shown in Supplementary Figure1 , no mediating effect of these three methylation factors was found.

## Discussion

The gender-specific association between maternal prenatal stress and offspring emotional and behavioral health has been fully summarized [39]. Studies from animal models indicated that gender-dimorphic alterations in placental glucocorticoid response genes to prenatal stress may provide the underlying mechanism [23, 24]. However, evidence from human studies is lacking. Our population-based prospective cohort study found that pregnancy-related anxiety at the third trimester was associated with emotional symptoms only in girls and hyperactivity only in boys of preschool age, which confirms and extends our previous research [7, 8]. Furthermore, only boys showed differential methylation of the *FKBP5* gene in response to prenatal pregnancy-related anxiety; also, methylation of *NR3C1* and *HSD11B2* genes was associated with the risk of emotional symptoms and hyperactivity only in boys. To the best of our knowledge, this is the first human study to explore whether gender-specific effects of maternal pregnancy-related anxiety during pregnancy on preschoolers’ neurobehavior depended upon the gender-dependent DNA methylation patterning of placental genes that regulate fetal exposure to glucocorticoids.

We add to the literature [39] by showing similar findings in pregnancy-related anxiety, a special mode of prenatal stress that has received little attention but predicts fetal development outcomes more accurately than general anxiety and depression symptoms [40, 41]. Compared with our two previous works [7, 8], the present study found similar gender-dependent outcomes but only focused on pregnancy-related anxiety at the third trimester because this cohort data showed that the third trimester is the critical period of pregnancy-related anxiety on child neurobehavioral development [42]. All the three works [7, 8] reported the male-bias hyperactivity symptoms but only two works [8] reported the female-bias emotional symptoms. In addition, a small sample size (*n* = 27) study showed that higher pregnancy-related anxiety symptoms were significantly associated with more emotional symptoms in boys compared to girls at 4 years old [31]. Thus, compared with the male-bias hyperactivity, the female-bias relationship of pregnancy-related anxiety with emotional symptoms needs to be further explored.

Our results partly support our hypothesis by finding that only boys showed differential methylation of the *FKBP5* gene in response to prenatal pregnancy-related anxiety; also, only in boys was the methylation of *NR3C1* and *HSD11B2* genes associated with the risk of emotional symptoms and hyperactivity, although no mediating effects were found. To date, there has been two human studies investigating a gender-specific association mechanism through the placental glucocorticoid signaling pathway [18, 43]. In Stroud et al. study, although the decrease of *HSD11B2* methylation in placenta was found to be associated with increased baseline cortisol in infants of mothers with prenatal major depression disorder, no gender difference was found in *HSD11B2* methylation in the placenta [18]. Besides, work from Green et al. that focused on American mothers suggested that the correlation between methylation and expression of the *HSD11B2* gene in human placenta with infant birth weight was found only in female and not male infants [43]. The role of placental methylation of genes regulating cortisol activity in the gender-specific association between prenatal stress and offspring outcomes needs to be further explored.

Pregnancy-related anxiety-induced DNA methylation of glucocorticoid regulatory genes may contribute to the neurological behaviour of children dependent on ethnicity or environment [29]. For example, a prospective cohort study with 509 American infants revealed that infants in the highest quartile of *FKBP5* methylation had increased risk of Network Neurobehavioral Scales (NNNS) high arousal compared to infants in the lowest quartile [11], but we found no association between *FKBP5* methylation and neurobehavioral outcomes in our present study, which focused on pregnant Chinese women. Furthermore, Grasso et al. found that infant saliva *FKBP5* methylation correlated with maternal post-traumatic stress disorder symptoms during pregnancy, but only in infants with the homozygous *FKBP5* rs1360780 C allele [44]. In addition, Capron et al. performed a multi-ethnic study and found that placental gene expression of *NR3C1* and *HSD11B2* is regulated by maternal prenatal anxiety and prenatal life events, but only in Caucasians, instead of South Asians and African/African-Americans [28]. Our data also showed no links between prenatal anxiety and placental *NR3C1* and *HSD11B2* methylation in Chinese. Thus, it is necessary to understand more about the role of social and biological differences in the mechnism of prenatal stress and psychopathology in the rest of the world, in addition to the majority of available research in Caucasians from high-income countries.

The current study had additional strengths that lend confidence to the findings. Firstly, this is a prospective cohort study with a good strength of causal reasoning, a relatively high response rate of participants and, by controlling multiple confounding factors (e.g. maternal and child characteristics and infant breastfeeding), thus the role of pregnancy-related anxiety on children’s development was detected more accurately. Secondly, pregnancy-related anxiety may be a more accurate predictor of adverse birth outcomes and child health than general anxiety [40]. Instead of using tools designed for the general population to assess pregnancy-related anxiety, such as the State-Trait Anxiety Inventory (STAI), our approach may be more accurate because it includes items specific to the mother’s experience during pregnancy [3]. Thirdly, since the gender-specific mechanism from the glucocorticoid signaling pathway in placenta has been well-documented [10, 14, 21, 22, 45, 46], our data may be more accurate compared to prior studies which have used fetal saliva or blooding of cortisol.

Although novel, this study has some limitations that should be taken into account. Firstly, we had no information of maternal postnatal mood and controlled it during our analysis, because the MABC Study only followed the development of the child, not the mother. However, maternal postnatal mood could be related to pregnancy-related anxiety and could affect child SDQ scores, like previous research [47]. Secondly, labor involves a series of complex hormonal processes including activation of placental corticotropin releasing hormone and glucocorticoid metabolism [48], although we controlled the mode of delivery, we did not distinguished elective caesarean from emergency caesarean. Thirdly, we used factor analysis, which is a statistical technique that use the idea of dimensionality reduction to describe variability among a number of measured, correlated variables as a lower number of latent factors, in order to reduce the number o f comparisons made. Although the cumulative contribution rate of the extracted 5 factors was relatively high (73.8%), the explained variance is small if many loci are combined in one factor. Lastly, there are also other important gender-specific modes of maternal–fetal stress transfer, such as placental inflammation [7], and additional research is required to determine how perturbations in stress-related biological responses conspire to influence poor offspring outcomes.

## Conclusions

In summary, we observed that the placental methylation of genes regulating glucocorticoids was associated with both maternal pregnancy-related anxiety and preschoolers’ emotional symptoms and hyperactivity in a gender-dependent manner. Combined with previous evidence, we think it is possible but not certain whether placental glucocorticoids genes methylation is the underlying mechanism because we did not find a mediating effect. Since the sample size for our mediation effect analysis is not large enough (all the power < 80%, see the Supplementary Figure 2), further verification with a larger sample size is required.

## Supplementary Information

**Additional file 1: Supplementary Figure 1.** A mediation model relating pregnancy-related anxiety in the third trimester, methylation of glucocorticoid regulation genes, and emotional symptoms and hyperactivity in 4 years old children. Factor 1 = latent methylation factor 1 was characterized by *NR3C1*; Factor 4 = latent methylation factor 4 was characterized by *HSD11B2*; Factor 5 = latent methylation factor 5 was characterized by *FKBP5*. The indirect effect of pregnancy-related anxiety on emotional symptoms and hyperactivity at age of 4 years old was not statistically significant.

**Additional file 2: Supplementary Figure 2.** The power of sex-specific mediating role of the latent methylation factors in the relationship between maternal pregnancy-related anxiety and children’s emotional symptoms and hyperactivity.

**Additional file 3: Supplemental Table 1.** Characteristics of participants and excluded participants.

**Additional file 4: Supplementary Table 2.** Characteristics of participants stratified by gender.

**Additional file 5: Supplementary Table 3.** Binary logistic regression models for associations between maternal pregnancy-related anxiety during the third trimester and emotional symptoms, hyperactivity in 4 years old children in DNA methylation samples.

## Data Availability

The datasets used and/or analysed during the current study are available from the corresponding author on reasonable request.

## Abbreviations

PNMS: Prenatal Maternal Psychological Stress
MABC: Ma’anshan Birth Cohort
SDQ: Strengths and Difficulties Questionnaire
TSS: Transcriptional Start Site
PCR: Polymerase Chain Reaction
BMI: Body Mass Index
OR: Odds Ratio
95% CI: 95% Confidence Interval
KMO: Kaiser-Meyer-Olkin
STAI: State-Trait Anxiety Inventory
NNNS: Network Neurobehavioral Scales
