# Supplementary material for: Gender-specific associations of pregnancy-related anxiety with placental epigenetic patterning of glucocorticoid response genes and preschooler’s emotional symptoms and hyperactivity
Source: BMC Pediatr. 2021 Oct 29;21:479. doi: 10.1186/s12887-021-02938-z (PMC8555194; doi:10.1186/s12887-021-02938-z)

Hui Liu et al. Gender-specific associations of pregnancy-related anxiety with placental epigenetic patterning of glucocorticoid response genes and preschooler's emotional symptoms and hyperactivity

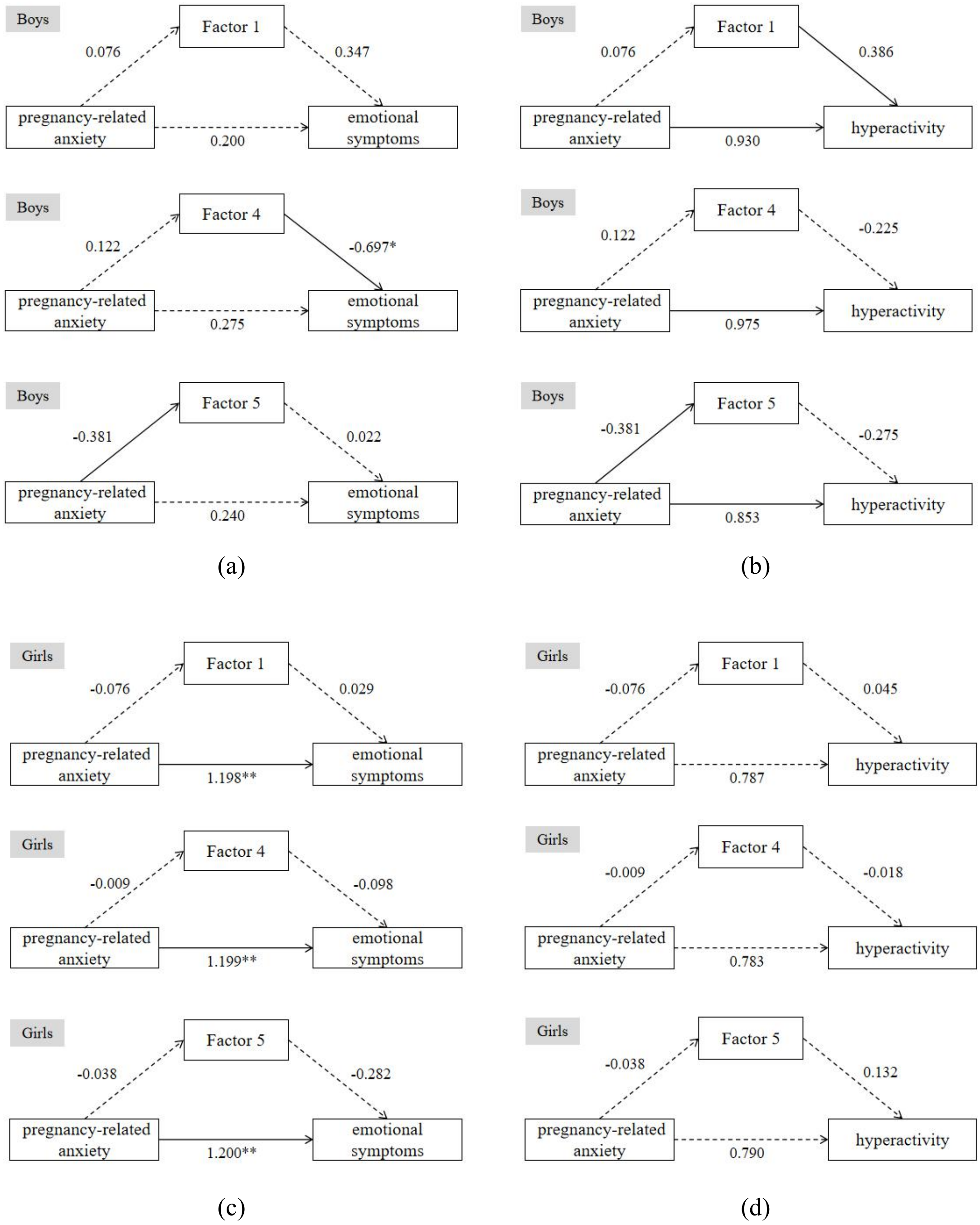

Supplement: Supplementary file 1 — Additional file 1: Supplementary Figure 1. A mediation model relating pregnancy-related anxiety in the third trimester, methylation of glucocorticoid regulation genes, and emotional symptoms and hyperactivity in 4 years old children. Factor 1 = latent methylation factor 1 was characterized by NR3C1; Factor 4 = latent methylation factor 4 was characterized by HSD11B2; Factor 5 = latent methylation factor 5 was characterized by FKBP5. The indirect effect of pregnancy-related anxiety on emotional symptoms and hyperactivity at age of 4 years old was not statistically significant. [file 12887_2021_2938_MOESM1_ESM.pdf]
