# Supplementary material for: Gender-specific associations of pregnancy-related anxiety with placental epigenetic patterning of glucocorticoid response genes and preschooler’s emotional symptoms and hyperactivity
Source: BMC Pediatr. 2021 Oct 29;21:479. doi: 10.1186/s12887-021-02938-z (PMC8555194; doi:10.1186/s12887-021-02938-z)
Supplement: Supplementary file 2 — Additional file 2: Supplementary Figure 2. The power of sex-specific mediating role of the latent methylation factors in the relationship between maternal pregnancy-related anxiety and children’s emotional symptoms and hyperactivity. [file 12887_2021_2938_MOESM2_ESM.pdf]

Hui Liu et al. Gender-specific associations of pregnancy-related anxiety with placental epigenetic patterning of glucocorticoid response genes and preschooler's emotional symptoms and hyperactivity

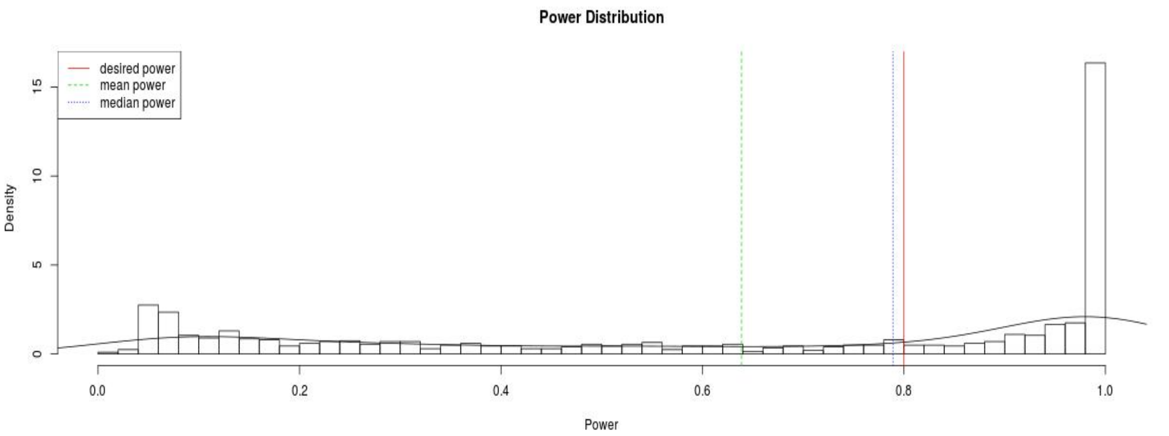

Summary Statistics of the Power Distribution

Show 25 entries Search:

MeanPower	MedianPower	Assurance
0.63871	0.789	0.493

The power of sex-specific mediating role of the latent methylation factor 1 in the relationship between maternal pregnancy-related anxiety and children's emotional symptoms in boys.

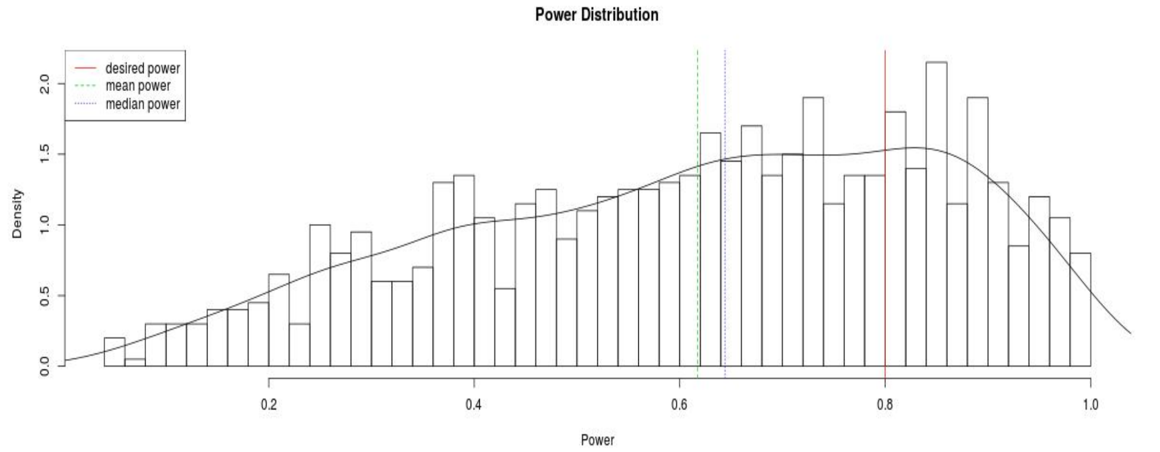

Summary Statistics of the Power Distribution

Show 25 entries Search:

MeanPower	MedianPower	Assurance
0.617504	0.644	0.273

The power of sex-specific mediating role of the latent methylation factor 4 in the relationship between maternal pregnancy-related anxiety and children's emotional symptoms in boys.

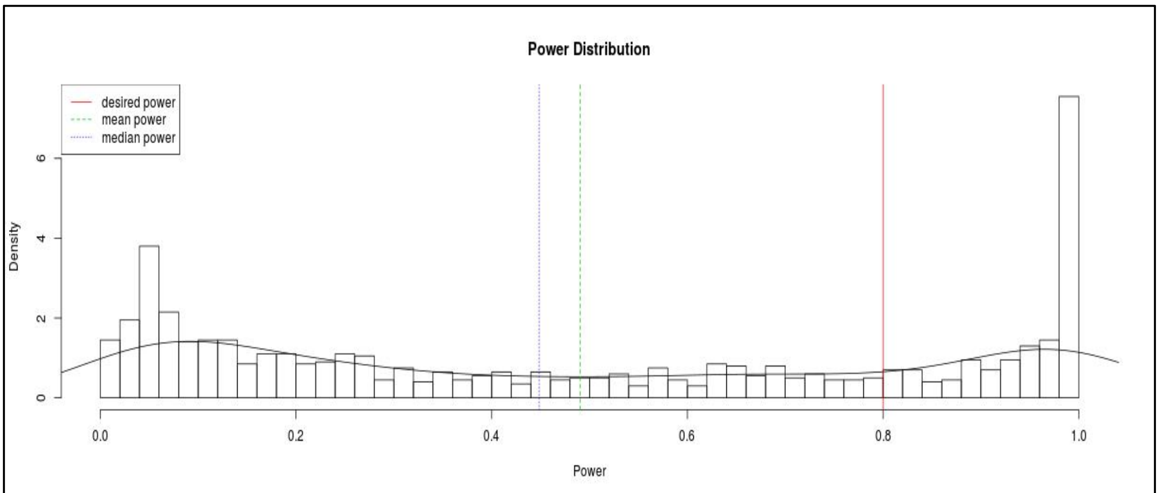

Summary Statistics of the Power Distribution

Show 25 entries

Search:

MeanPower	MedianPower	Assurance
0.49035	0.4485	0.304

The power of sex-specific mediating role of the latent methylation factor 5 in the relationship between maternal pregnancy-related anxiety and children's emotional symptoms in boys.

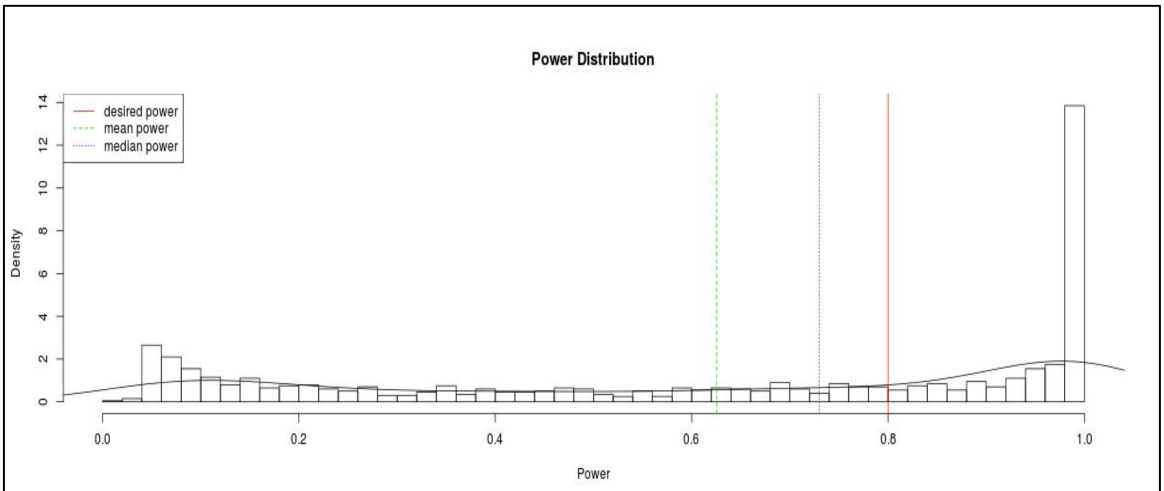

Summary Statistics of the Power Distribution

Show 25 entries

Search:

MeanPower	MedianPower	Assurance
0.625746	0.73	0.453

The power of sex-specific mediating role of the latent methylation factor 1 in the relationship between maternal pregnancy-related anxiety and children's hyperactivity in boys.

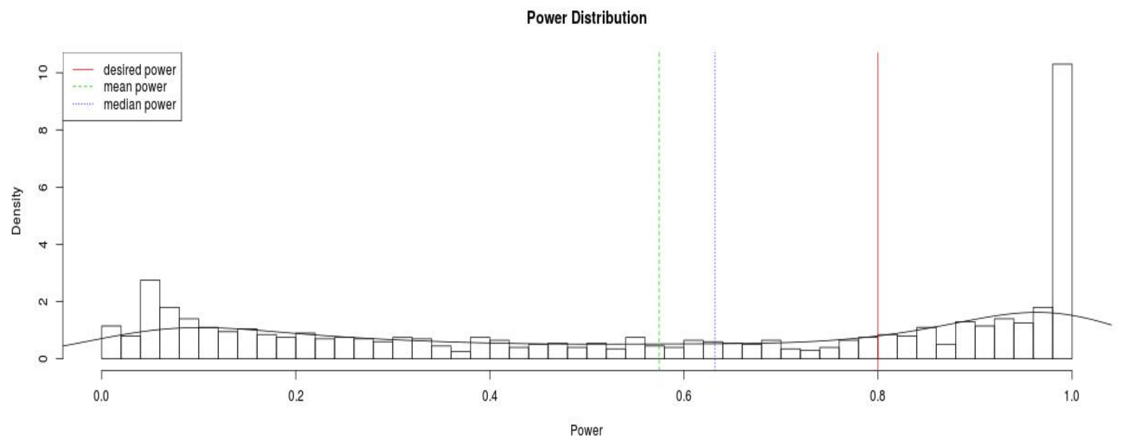

Summary Statistics of the Power Distribution

Show 25 entries

Search:

MeanPower	MedianPower	Assurance
0.574518	0.632	0.409

The power of sex-specific mediating role of the latent methylation factor 4 in the relationship between maternal pregnancy-related anxiety and children's hyperactivity in boys

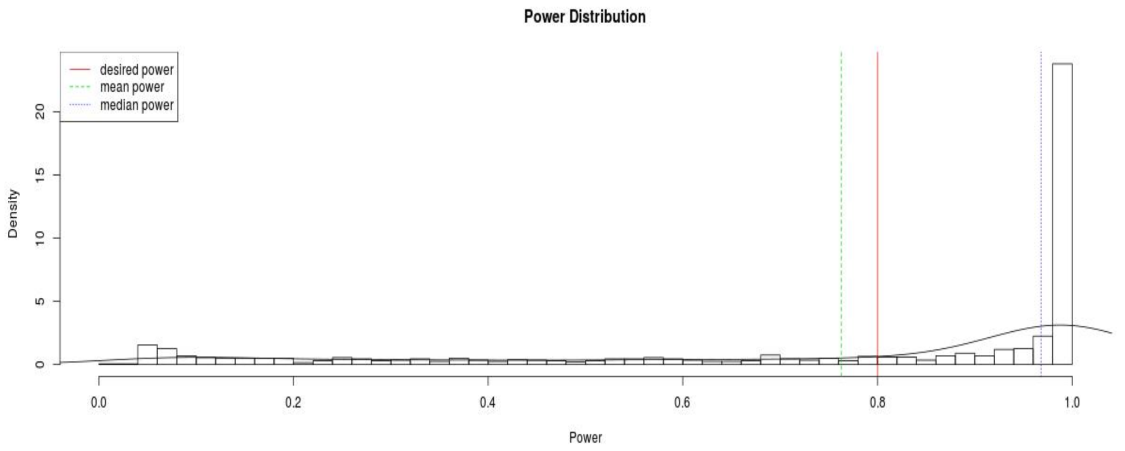

Summary Statistics of the Power Distribution

Show 25 entries

Search:

MeanPower	MedianPower	Assurance
0.762951	0.968	0.648

The power of sex-specific mediating role of the latent methylation factor 5 in the relationship between maternal pregnancy-related anxiety and children's hyperactivity in boys

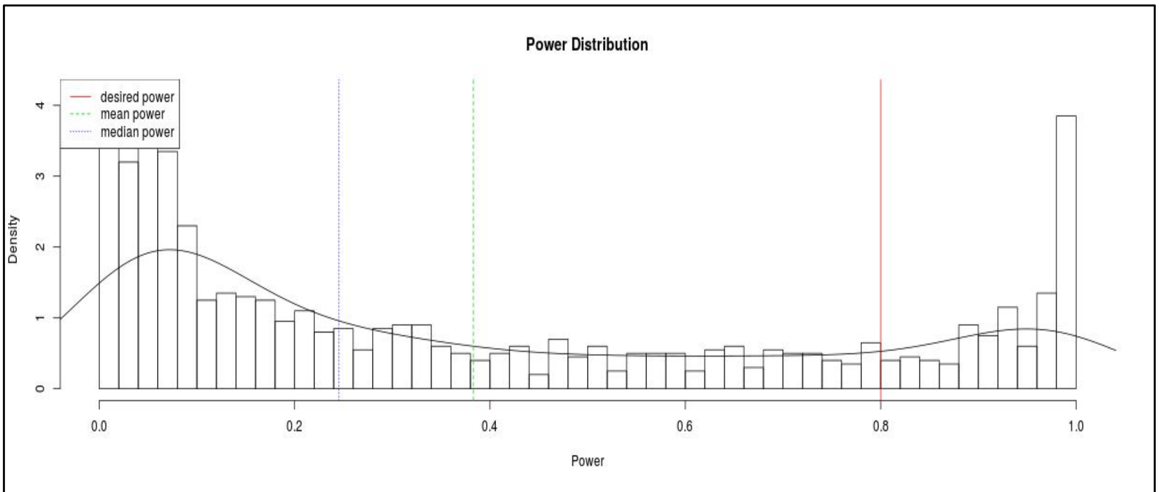

Summary Statistics of the Power Distribution

Show 25 entries Search:

MeanPower	MedianPower	Assurance
0.382907	0.2455	0.205

The power of sex-specific mediating role of the latent methylation factor 1 in the relationship between maternal pregnancy-related anxiety and children's emotional symptoms in girls.

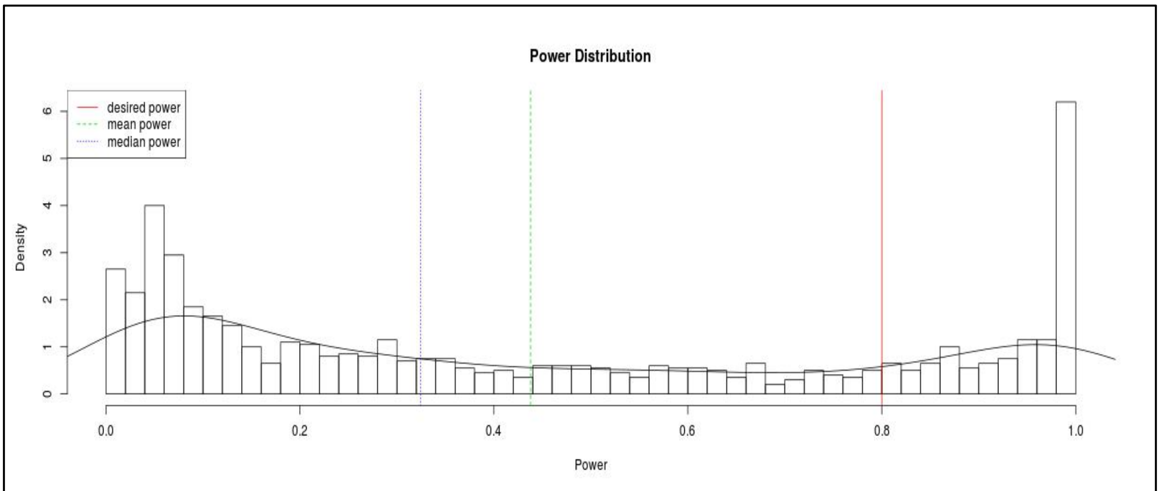

Summary Statistics of the Power Distribution

Show 25 entries Search:

MeanPower	MedianPower	Assurance
0.43784	0.3245	0.266

The power of sex-specific mediating role of the latent methylation factor 4 in the relationship between maternal pregnancy-related anxiety and children's emotional symptoms in girls.

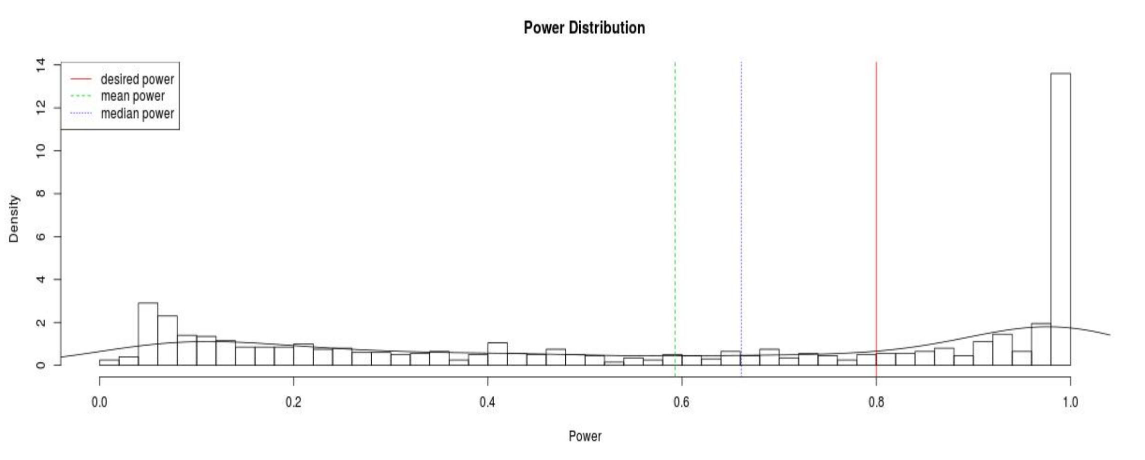

Summary Statistics of the Power Distribution

Show 25 entries Search:

MeanPower	MedianPower	Assurance
0.592821	0.661	0.436

The power of sex-specific mediating role of the latent methylation factor 5 in the relationship between maternal pregnancy-related anxiety and children’s emotional symptoms in girls.

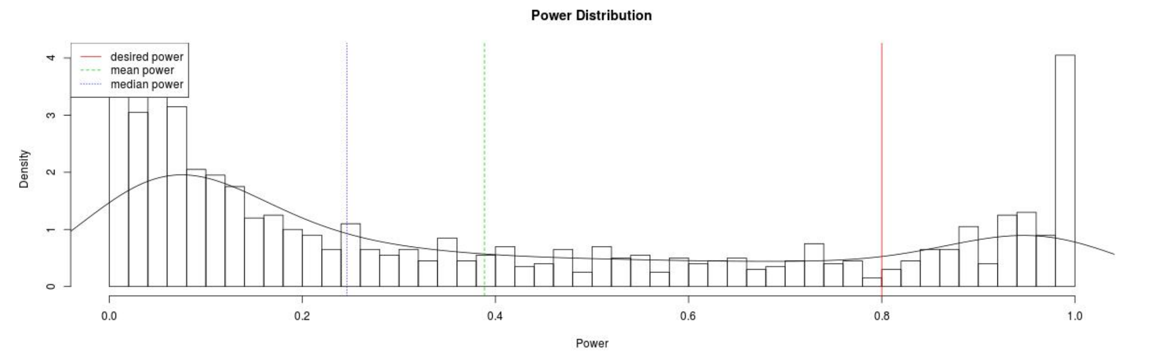

Summary Statistics of the Power Distribution

Show 25 entries Search:

MeanPower	MedianPower	Assurance
0.388598	0.246	0.22

The power of sex-specific mediating role of the latent methylation factor 1 in the relationship between maternal pregnancy-related anxiety and children’s hyperactivity in girls

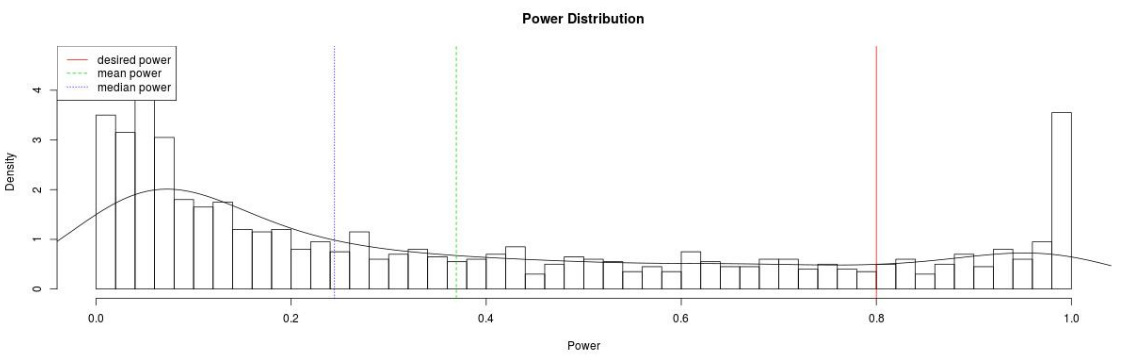

Summary Statistics of the Power Distribution

Show 25 entries Search:

MeanPower	MedianPower	Assurance
0.369344	0.2445	0.179

The power of sex-specific mediating role of the latent methylation factor 4 in the relationship between maternal pregnancy-related anxiety and children's hyperactivity in girls

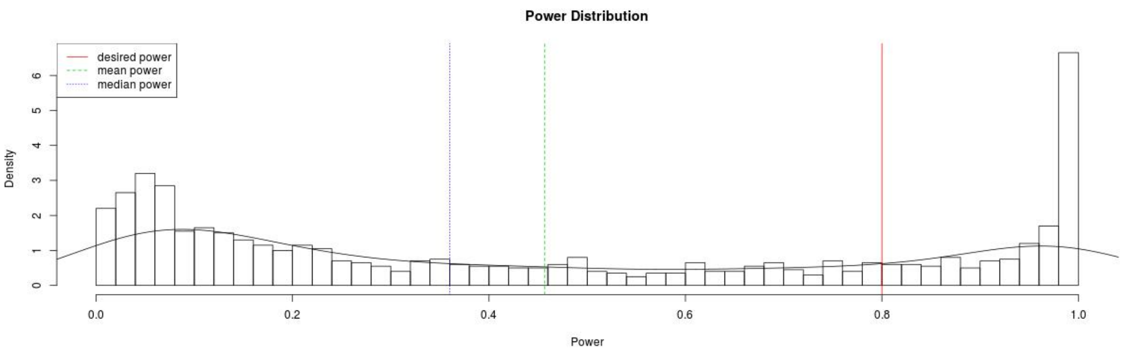

Summary Statistics of the Power Distribution

Show 25 entries Search:

MeanPower	MedianPower	Assurance
0.456857	0.36	0.282

The power of sex-specific mediating role of the latent methylation factor 5 in the relationship between maternal pregnancy-related anxiety and children's hyperactivity in girls
