# Supplementary material for: Gender-specific associations of pregnancy-related anxiety with placental epigenetic patterning of glucocorticoid response genes and preschooler’s emotional symptoms and hyperactivity
Source: BMC Pediatr. 2021 Oct 29;21:479. doi: 10.1186/s12887-021-02938-z (PMC8555194; doi:10.1186/s12887-021-02938-z)
Supplement: Supplementary file 3 — Additional file 3: Supplemental Table 1. Characteristics of participants and excluded participants. [file 12887_2021_2938_MOESM3_ESM.docx]

| **Supplemental table 1** Characteristics of participants and excluded participants | | |
| --- | --- | --- |
|  | Participants | Excluded participants |
| Maternal age, years | 26.63±3.60 | 26.54±3.76 |
| Pre-pregnancy BMI**^*^** | 20.58±2.77 | 20.67±2.83 |
| Gestational weight gain**^*^** | 17.72±5.06 | 18.13±5.18 |
| Maternal education |  |  |
| Bachelor degree or above | 641(26.7) | 221(25.5) |
| Junior college | 753(31.3) | 263(30.3) |
| Senior high school or equal | 542(22.5) | 193(22.2) |
| Junior high school or below | 469(19.5) | 191(22.0) |
| Family monthly income |  |  |
| <2500 RMB | 670(27.9) | 197(22.7) |
| 2500-4000 RMB | 1020(42.4) | 382(44.0) |
| >4000 RMB | 715(29.7) | 289(33.3) |
| Maternal smoking |  |  |
| Never | 2308(96.0) | 823(94.8) |
| Former/current | 97(4.0) | 45(5.2) |
| Maternal drinking |  |  |
| Never | 2218(92.2) | 794(91.5) |
| Occasionally/frequently | 187(7.8) | 74(8.5) |
| Gestational age at delivery | 39.10±1.24 | 38.82±1.70 |
| Parity |  |  |
| Primipara | 2152(89.5) | 746(85.9) |
| Multipara | 253(10.5) | 122(14.1) |

**Hui Liu et al. Gender-specific associations of pregnancy-related anxiety with placental epigenetic patterning of glucocorticoid response genes and preschooler’s emotional symptoms and hyperactivity**

Abbreviations: BMI, body max index. RMB;Chinese monetary unit *yuan*.

^*^The survey data is missing.
