# Supplementary material for: Gender-specific associations of pregnancy-related anxiety with placental epigenetic patterning of glucocorticoid response genes and preschooler’s emotional symptoms and hyperactivity
Source: BMC Pediatr. 2021 Oct 29;21:479. doi: 10.1186/s12887-021-02938-z (PMC8555194; doi:10.1186/s12887-021-02938-z)
Supplement: Supplementary file 4 — Additional file 4: Supplementary Table 2. Characteristics of participants stratified by gender. [file 12887_2021_2938_MOESM4_ESM.docx]

**Hui Liu et al. Gender-specific associations of pregnancy-related anxiety with placental epigenetic patterning of glucocorticoid response genes and preschooler’s emotional symptoms and hyperactivity**

| **Sulplementary table 2** Characteristics of participants stratified by gender | | | | |
| --- | --- | --- | --- | --- |
|  | Prenatal anxiety | Control | *χ^2^/t* | *P* |
| Boys |  |  |  |  |
| Maternal age, years | 26.33±3.24 | 26.89±3.85 | 2.41 | **0.010** |
| Gestational weight gain^*^ | 18.34±4.92 | 17.41±5.11 | -2.94 | **0.003** |
| Maternal education |  |  |  |  |
| Bachelor degree or above | 84(23.3) | 243(27.8) | 3.73 | 0.293 |
| Junior college | 113(31.4) | 277(31.7) |  |  |
| Senior high school or equal | 90(25.5) | 185(21.2) |  |  |
| Junior high school or below | 73(20.3) | 169(19.3) |  |  |
| Maternal smoking |  |  | 3.71 | 0.054 |
| Never | 339(94.2) | 844(96.6) |  |  |
| Former/current | 21(5.8) | 30(3.4) |  |  |
| Caesarean^*^ | 199(55.4) | 410(41.7) | 7.11 | **0.008** |
| Exclusive breastfeeding at first 6 months^*^ | 31(8.8) | 109(12.7) | 3.90 | **0.048** |
| Girls |  |  |  |  |
| Maternal age, years | 26.08±3.39 | 26.73±3.53 | 2.94 | **0.003** |
| Gestational weight gain^*^ | 17.91±5.10 | 17.70±5.02 | -0.65 | 0.518 |
| Maternal education |  |  |  |  |
| Bachelor degree or above | 79(22.4) | 235(28.7) | 8.37 | **0.039** |
| Junior college | 104(29.5) | 259(31.7) |  |  |
| Senior high school or equal | 92(26.1) | 175(21.4) |  |  |
| Junior high school or below | 78(22.1) | 149(18.2) |  |  |
| Maternal smoking |  |  | 7.11 | **0.008** |
| Never | 331(93.8) | 794(97.1) |  |  |
| Former/current | 22(6.2) | 24(2.9) |  |  |
| Caesarean^*^ | 190(54.3) | 409(50.2) | 1.60 | 0.206 |
| Exclusive breastfeeding at first 6 months^*^ | 28(8.2) | 106(13.2) | 5.87 | **0.015** |

Abbreviations: ^*^The survey data is missing.
