# Supplementary material for: Gender-specific associations of pregnancy-related anxiety with placental epigenetic patterning of glucocorticoid response genes and preschooler’s emotional symptoms and hyperactivity
Source: BMC Pediatr. 2021 Oct 29;21:479. doi: 10.1186/s12887-021-02938-z (PMC8555194; doi:10.1186/s12887-021-02938-z)
Supplement: Supplementary file 5 — Additional file 5: Supplementary Table 3. Binary logistic regression models for associations between maternal pregnancy-related anxiety during the third trimester and emotional symptoms, hyperactivity in 4 years old children in DNA methylation samples. [file 12887_2021_2938_MOESM5_ESM.docx]

**Hui Liu et al. Gender-specific associations of pregnancy-related anxiety with placental epigenetic patterning of glucocorticoid response genes and preschooler’s emotional symptoms and hyperactivity**

| **Sulplementary table3** Binary logistic regression models for associations between maternal pregnancy-related anxiety during the third trimester and emotional symptoms, hyperactivity in 4 years old children in DNA methylation samples | | | | | | | | | |
| --- | --- | --- | --- | --- | --- | --- | --- | --- | --- |
| Prenatal-related anxiety | Emotional Symptoms | | | |  | Hyperactivity | | | |
|  | Normal | Borderline and Abnormal | OR^a^(95%CI) | *P*^a^ |  | Normal | Borderline and Abnormal | OR^a^(95%CI) | *P*^a^ |
| Total samples |  |  |  |  |  |  |  |  |  |
| No | 151(85.3) | 26(14.7) | 1.00 |  |  | 157(88.7) | 20(11.3) | 1.00 |  |
| Yes | 128(74.4) | 44(25.6) | 1.40(0.76,2.59) | 0.285 |  | 132(76.7) | 40(23.3) | 1.89(1.00,3.58) | **0.049** |
| Boys |  |  |  |  |  |  |  |  |  |
| No | 69(81.2) | 16(18.8) | 1.00 |  |  | 73(85.9) | 12(14.1) | 1.00 |  |
| Yes | 66(77.6) | 19(22.4) | 0.77(0.31,1.91) | 0.566 |  | 60(70.6) | 25(29.4) | 1.98(0.81,4.85) | 0.134 |
| Girls |  |  |  |  |  |  |  |  |  |
| No | 82(89.1) | 10(10.9) | 1.00 |  |  | 84(91.3) | 8(8.7) | 1.00 |  |
| Yes | 62(71.3) | 25(28.7) | 2.45(0.96,6.28) | 0.061 |  | 72(82.8) | 15(17.2) | 1.72(0.62,4.78) | 0.301 |

Abbreviations: CI, confidence interval; OR, odds ratio.

^a^ represents that these ORs adjusted by maternal age, pre-pregnancy BMI, Gestational weight gain, education, family monthly income, smoking, drinking, gestational diabetes, pregnancy-induced hypertension, delivery mode and exclusive breastfeeding at first 6 months
